# Changes in intestinal flora of mice induced by rEg.P29 epitope peptide vaccines

**DOI:** 10.1002/iid3.1082

**Published:** 2023-11-22

**Authors:** Tingting Zhang, Yongxue Lv, Yinqi Zhao, Jihui Yang, Bingshuo Qian, Yazhou Zhu, Wei Zhao, Mingxing Zhu

**Affiliations:** ^1^ School of Clinical Medicine Ningxia Medical University Yinchuan China; ^2^ Key Laboratory of Common Infectious Disease Prevention and Control in Ningxia Yinchuan China; ^3^ School of Basic Medical Sciences Ningxia Medical University Yinchuan China; ^4^ Science and Technology Center of Ningxia Medical University Yinchuan China; ^5^ General Hospital of Ningxia Medical University Yinchuan China

**Keywords:** 16 S rRNA sequencing, cystic echinococcosis, *Echinococcus granulosus*, intestinal flora, peptide vaccine

## Abstract

**Objective:**

Cystic echinococcosis (CE), a zoonotic parasitic disease caused by *Echinococcus granulosus*, remains a public health and socioeconomic issue worldwide, making its prevention and treatment of vital importance. The aim of this study was to investigate changes in the intestinal microbiota of mice immunized with three peptide vaccines based on the recombinant antigen of *E. granulosus*, P29 (rEg.P29), with the hope of providing more valuable information for the development of vaccines against CE.

**Methods:**

Three peptide vaccines, rEg.P29_T_, rEg.P29_B_, and rEg.P29_T + B_, were prepared based on rEg.P29, and a subcutaneous immunization model was established. The intestinal floras of mice in the different immunization groups were analyzed by 16 S rRNA gene sequencing.

**Results:**

The intestinal microbiota analysis at both immunization time points revealed that Firmicutes, Bacteroidota, and Verrucomicrobiota were the predominant flora at the phylum level, while at the genus level, Akkermansia, unclassified_Muribaculaceae, Lachnospiraceae_NK4A136_group, and uncultured_rumen bacterium were the dominant genera. Some probiotics in the intestines of mice were significantly increased after immunization with the peptide vaccines, such as Lactobacillus_taiwanensis, Lactobacillus_reuteri, Lachnospiraceae_NK4A136_group, Bacteroides_acidifaciens, and so forth. Meanwhile, some harmful or conditionally pathogenic bacteria were decreased, such as Turicibacter sanguinis, Desulfovibrio_fairfieldensis, Clostridium_sp, and so forth, most of which are associated with inflammatory or infectious diseases. Kyoto Encyclopaedia of Genes and Genomes enrichment analysis revealed that the differential flora were enriched in multiple metabolic pathways, primarily biological systems, human diseases, metabolism, cellular processes, and environmental information processing.

**Conclusion:**

In this study, we comprehensively analyzed and compared changes in the intestinal microbiota of mice immunized with three peptide vaccines as well as their related metabolic pathways, providing a theoretical background for the development of novel vaccines against *E. granulosus*.

## Graphical abstract

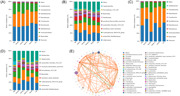

## 1 INTRODUCTION

Cystic echinococcosis (CE) is a zoonotic parasitic disease caused by *Echinococcus granulosus*, which is more prevalent in countries and regions with well‐developed animal husbandry and causes serious health and economic loss.^1^, ^2^ It is particularly prevalent in Central Asia, western China, southern Europe, northern and central Africa, and south‐western Latin America.^3^ In China, it is predominantly endemic to the western region, including Xinjiang, Ningxia, Qinghai, Gansu, and Tibet.^4^ According to the latest update from the World Health Organization (WHO) in March of 2017, the number of infected cases of echinococcosis could exceed one million at any given time, demonstrating that the current epidemiological form of *E. granulosus* remains a serious concern. The life history of *Echinococcus* involves two groups of mammals, with canids (i.e., dogs and foxes) as the final host and herbivores (i.e., sheep and cattle), as well as humans, as intermediate hosts. Humans, sheep, and other intermediate hosts may develop echinococcosis after accidental ingestion of *E. granulosus* eggs.^5^, ^6^ Although various treatments are available for different stages of infection, such as percutaneous, surgical, and pharmacological treatments, the risk of recurrence remains a major problem.^7^, ^8^ Widely used pharmacological treatments are often based on anthelmintic drugs such as albendazole and praziquantel.^9^ While a cure has not yet been discovered, these drugs are used preoperatively and postoperatively as a therapeutic tool to prevent metastatic recurrence. Although imaging techniques are a routine means of diagnosing CE, they do not effectively detect the infection at an early stage because the liver worm exhibits a slow incubation period of 10–20 years in the human body.^10^ As a result, the development of vaccines has emerged as a more reliable solution for reducing the prevalence of CE.

Recently, genetically engineered vaccines have received more attention in studies of early prevention. Some reported data showed that a vaccine derived from the Eg95 protein shows 96–98% protection in sheep.^11^ In addition, other novel vaccines are being developed, including those based on the P29 protein, a new 29 kDa antigen first identified by Gonzalez et al. from the protocephalic nodes of *E. granulosus*.^12^ As a diagnostic antigen, it has high sensitivity and specificity.^13^ The pET28a‐P29/BL21 strain, which was constructed and preserved in our laboratory, was sequenced, confirmed to be free of mutation, and purified by nickel column affinity chromatography using isopropyl‐β‐d‐thiogalactoside (IPTG)‐induced expression to obtain the recombinant protein of *E. granulosus* P29 (rEg. P29). It was found to be 96.6% and 94.5% immunoprotective in mice and sheep, respectively, by postimmunisation reinfection experiments.^14^ It shows potential as a candidate vaccine against *E. granulosus* infection. However, due to the limitation of the expression system, this genetically engineered vaccine is difficult to mass produce and contains an endotoxin that is difficult to remove. Therefore, finding a more efficient, broad‐spectrum, cheap, and safe vaccine is pertinent. Peptide vaccines, which use antigenic epitopes to trigger immunity, are a relatively new type of vaccine. Most are synthetic and do not contain nucleic acids, allowing them to overcome some of the shortcomings of conventional vaccines, including improved efficiency and high specificity.^15^ Here, we prepared three peptide vaccines, rEg.P29_T_, rEg.P29_B_, and rEg.P29_T + B_, based on rEg.P29. Using a subcutaneous immunization model, we found through extensive experiments that immunization of mice with the peptide vaccines induced T helper cell type 1 (Th1)‐mediated cellular immune responses, leading to high levels of rEg.P29 or rEg.P29_B_ specific antibodies. Furthermore, the rEg.P29_T + B_ vaccine induced higher levels of antibody and cytokine production and longer immunological memory than the monoepitope vaccines.^16^ Its protective power was verified by postimmunisation reinfection experiments, which revealed that the cyst inhibition rate was significantly higher in mice immunised with the three peptides,     and the HE stained sections also revealed that the livers of mice from the peptide‐immunized group Inflammatory cell infiltration was significantly reduced, and ELISA results also showed that mice produced a large amount of Th1‐type cytokines. (Data not published). This evidence strongly suggests that peptide vaccines are more advantageous than protein vaccines.

With growing attention in recent years, gut microbiota has a significant impact on human health, mainly through regulating digestion, physiology, nutrition, and immunity. Dysbiosis of intestinal flora can disturb the regulation of the body's immune system, damage the intestinal mucosal barrier, and induce chronic inflammatory diseases. Different gut bacteria may be involved in the differentiation and functional regulation of different immune cell subpopulations.^17^ Nicaise et al. suggested that intact gut flora provides a basis for the production of interleukin (IL)‐12 by the spleen, an important part of both innate and acquired immunity. IL‐12 effectively improves cellular immune defences and promotes the differentiation of CD4^+^ T‐cells to Th1 cells.^18^ It has been reported that gut microbes can directly induce a proinflammatory response by enhancing the production of proinflammatory cytokines and/or polarizing naïve T cells towards Th1 and Th17 phenotypes, which directly sensitize gut epithelial‐associated antigen‐presenting cells. Metabolites released by gut microbes can also induce a regulatory T cell (Treg) response. This Treg response plays a crucial role in suppressing inflammatory responses caused by pathogenic infections or inflammatory diseases/autoimmunity and also maintains immune homeostasis in the gut. At the same time, the induced protective effect produced by gut microbes against parasitic infections is due to the release of short‐chain fatty acids (SCFA) (e.g., butyrate), which block M1 macrophage polarization and DC activation to inhibit infections by different parasites (e.g. Plasmodium, Giardia and Toxoplasma gondii).^19^, ^20^, ^21^ Beneficial gut bacteria have many important functions, including producing a variety of nutrients for the host, preventing infections caused by intestinal pathogens, and regulating normal immune responses.^22^ Numerous investigations have reported that probiotics can exert a wide range of therapeutic effects including modulation of immunity, prevention of cancer, and prevention or reduction of atopic dermatitis and diarrhoea, among other effects. Also, when the reduced diversity of gut microbes creates an imbalance between anti‐inflammatory and proinflammatory cellular responses leading to intestinal inflammation, the use of probiotic microbes can reduce this imbalance and ultimately ameliorate inflammatory diseases.^23^ And it has also been found that for various cancers associated with the digestive system as well as various intestinal infectious diseases, prebiotics reduce inflammation associated with the intestinal tract primarily by increasing the beneficial microbiota in the gut (such as Bifidobacteria and Lactobacillus) and by producing SCFA. In addition, it is useful in preventing infectious agents from entering the gut.^24^ A recent study on immunosuppressed mice showed that injections of *Lactobacilli* enhanced intestinal resistance to *Cryptosporidium microsporum*.^25^ As our understanding of how the gut microbiota functions grows, promising solutions have been found in the treatment of diseases, such as probiotics, drugs, and fecal transplants, which have greatly improved the state of human health.^26^ Of course, colonization by pathogenic bacteria can also lead to microecological dysregulation of the organism, which is associated with many life‐threatening human diseases. For example, intestinal pathogens damage the protective intestinal mucosal layer leading to inflammation, tissue damage, reactive oxygen species generation and tumor formation. Several studies have reported that alterations in gut microbial homeostasis as well as pathogenic bacteria may trigger the oncogenic process of colorectal cancer (CRC). Among them, F. nucleatum is considered to be one of the pathogenic bacteria that induce CRC in humans.^27^ Previous studies in our laboratory also found increased abundance of Firmicutes and Proteobacteria in mice infected with *E. granulosus* compared to the normal group.^28^

Over the past few years, several studies have focused on the relationship between parasitic infections and the gut microbiota, showing that parasitic infections affect the composition of the host gut flora, while the gut flora also affects the viability and infectivity of the parasite.^29^ The most common site of *E. granulosus* infection is the liver, where changes in the gut microbiota can have a dramatically affect as intestinal bacteria or their metabolites enter the liver via the portal vein.^30^ However, there are few studies to date on the interplay between the gut microbiota and *E. granulosus* infections. Liu et al.^31^ reported that *E. granulosus* infection reduced the diversity and percentage of beneficial bacteria in the gut microbiota of Tibetan sheep. Bao et al.^32^ used a mouse model to study the effects of chronic *E. granulosus* infections on the gut microbiota. They found that the guts of infected mice were enriched in two genera, *Eisenbergiella* and *Parabacteroids*. These findings provide potential new strategies for the control of *E. granulosus*. Therefore, in this study, we prepared three peptide vaccines, rEg.P29_T_, rEg.P29_B_, and rEg.P29_T + B_, based on rEg.P29, established a subcutaneous immunity model using mice, and carried out 16 S rRNA sequencing of the intestinal flora to compare and analyze the variation in the gut microbiota between the groups, providing a basis for the development of vaccines against *E. granulosus*.

## 2 MATERIALS AND METHODS

### 2.1 Experimental animals

Female 6–8‐week‐old C57BL/6 mice, weighing approximately 18–25 g each, were purchased from the Experimental Animal Center of Ningxia Medical University. All mice were placed in a specific‐pathogen free environment at 22°C for 1 week before the experiment. Animal experiments were performed in strict accordance with the Guide for the Management of Laboratory Animals and the National Institute of Health Guidelines for the Care and Use of Laboratory Animals and approved by the Animal Ethics Committee of Ningxia Medical University.

### 2.2 Protein purification and vaccine preparation

In the preliminary stage of this research group, we extracted the RNA of *E. granulosus* protocephalicus, PCR amplified the P29 gene of *E. granulosus*, and then recombinant expression vector was constructed by recombining it into pET28a vector, finally, the constructed expression vector EgP‐29/pET28a was transformed into *Escherichia coli* BL21.^14^ Protein expression was induced by 50 μg/mL isopropyl‐β‐d‐thiogalactopyranoside (IPTG; Invitrogen) for 12 h. Since the recombinant P29 protein vector was constructed with a His tag added to its N‐terminus, purification was carried out using the principle of binding of His to Ni ions., rEg.P29 proteins were purified using a His purification kit (Merck), and the endotoxin was subsequently removed using an endotoxin removal kit (Genscript). Then, proteins were filtered using a 0.22‐μm sterilization filter to obtain rEg.P29 that met the experimental criteria. Epitope peptide screening was carried out by the overlapping peptide method, and the final screening yielded the dominant T‐cell epitope (rEg.P29_86‐100_ [ALEQVASQSEKAAPQ]) and B‐cell epitope (rEg.P29_166‐185_ [LKNAKTAEQKAKWEAEVRKD]) of rEg.P29.^16^ The synthetic peptides were submitted to Sangon Biotech (Shanghai, China) for synthesis, and three epitope peptide vaccines (rEg.P29_T_, rEg.P29_B_, and rEg.P29_T + B_) were obtained by coupling carrier proteins (keyhole limpet hemocyanin, KLH) to the screened T‐ and B‐cell epitope peptides. The rEg.P29_T + B_ was constructed by coupling the T‐cell epitope peptide and the B‐cell epitope peptide to the KLH vector at the N‐terminal end of the B‐cell epitope peptide after linking the two peptides using a universal linker, GSGSGS, to obtain a combined T‐cell and B‐cell epitope peptide.^16^ The adjuvant CpG ODN 1826 (TCCAT GAC GTT CCT GACGTT) was synthesized by Sangon.

### 2.3 Immunization modeling

Thirty female C57BL/6 mice were randomly divided into six groups: the phosphate buffer solution (PBS), rEg.P29_T_ + CpG, rEg.P29_B_ + CpG, rEg.P29_T + B_ + CpG, rEg.P29+CpG, and CpG groups (*n* = 5). Each mouse was immunized with 20 μg rEg.P29_T_, rEg.P29_B_, rEg.P29_T + B_ or rEg.P29 mixed with 40 μg CpG. The mixtures were diluted with PBS (100 μL), and the mice were immunized by subcutaneous triple injection via the abdomen, the first booster vaccination was carried out at an interval of 1 week after the first immunization, and the second booster vaccination was carried out at an interval of 3 weeks after the first booster vaccination.

### 2.4 Sample preparation and library sequencing

The feces of mice were collected 2 and 16 weeks after the second booster immunization, placed in freezing tubes, and stored at −80°C for subsequent use. First, DNA extraction of the samples was carried out (TIANGEN, DP328), following the steps of TIANGEN Fecal Genomic DNA Extraction Kit. The samples were lysed by weighing 180−220 mg of fecal sample into a 2 mL centrifuge tube, and the lysate was added to the samples along with Proteinase K and grinding beads and mixed by shaking, and incubated at 37°C for 15 min. Centrifuged at 12,000 rpm for 3 min, transferred the supernatant to a new centrifuge tube, added RNase A, shaking and mixing, and then left at room temperature for 5 min, added an equal volume of buffer, added the resulting solution to an adsorbent column, centrifuged at 12,000 rpm for 30 s, poured off the waste solution, and placed the column into a collection tube. Next, 700 μl of rinse solution was added to the adsorbent column, centrifuged at 12,000 rpm for 30 s, the waste liquid was poured off, and the column was allowed to stand at room temperature for a few minutes to thoroughly dry out the residual rinse solution in the adsorbent material. Finally, ultrapure water was used to dissolve the DNA sample on the adsorbent column membrane (approximately 50 μL). After the sample DNA extraction was completed, the nucleic acid concentration was detected using an enzyme labeller (manufacturer: GeneCompangLimited, model: synergyHTX). The amplified PCR products were detected by electrophoresis using agarose at a concentration of 1.8% (manufacturer: Beijing Bomei Fuxin Science and Technology Co., Ltd.), and the concentration and purity of DNA were determined using a NanoDrop2000 (ThermoScientific). The library construction and sequencing, a two‐step process, first uses DNA as the template and primers with junctions for PCR and then uses the PCR products from the first step as the template for PCR. The purpose of the primer junctions is to facilitate the addition of the barcode/index in the second step of the library construction, which is used to differentiate between bases of the samples. The sequencing is performed using the Illumina Novaseq. 6000 platform.

### 2.5 Diversity analysis

Circular consensus sequencing (CCS) sequences were derived from the raw data, and barcode identification, length filtering, and chimera removal were performed on the CCS sequences to obtain effective CCS sequences. Clustering/de‐noising of the effective CCS sequences was performed to classify the operational taxonomic units (OTUs). Next, the alpha diversity indices of the samples were evaluated using QIIME2 software. Differences in alpha diversity indices between groups were assessed using a *t*‐test. Beta diversity analysis was performed using QIIME software to compare the degree of similarity in species diversity present in the different samples. This analysis was performed using four algorithms: “binary jaccard,” “bray curtis,” “weighted unifrac,” and ”unweighted unifrac.”

### 2.6 Species annotation analysis

SILVA was used as the reference database to annotate the taxonomies of the feature sequences using a simple Bayesian classifier combined with a comparison method to obtain the taxonomic information of the species corresponding to each feature. Then, the community composition of each sample was counted at each taxonomic level (phylum, class, order, family, genus, and species). The abundance tables of the species at different taxonomic levels were generated by using QIIME software, and the community structure of the samples was plotted using the R tool under the various taxonomic levels.

### 2.7 Metastats analysis

The species abundance data between groups were subjected to a *t*‐test using Metastats software. *p* Values were obtained and used to filter out the species that contributed to the differences in the composition of the samples from the two groups.

### 2.8 Functional predictive analytics

The PICRUSt2 software was used to annotate the feature sequences to be predicted with the existing phylogenetic tree, and the IMG microbial genome data was used to output functional information, allowing inference of the functional gene composition of the samples, so as to analyze the differences in function between different samples or subgroups. Functional abundance between samples was tested for significant differences between two samples using the G‐TEST (large samples: number of functional genes annotated > 20) and Fisher (small samples: number of functional genes annotated < 20) tests in STAMP and between groups using a two‐by‐two *T*‐test with a *p* Value threshold of .05 (<.05 indicates statistical significance). The composition and difference analysis of Kyoto Encyclopaedia of Genes and Genomes (KEGG) metabolic pathways highlighted variations in metabolic pathways of functional genes of microbial communities between samples of different subgroups.

### 2.9 Data analysis

All data were analyzed and presented as mean ± standard deviation using GraphPad Prism 8.0 software. Independent samples *t*‐tests were used to compare the means between two groups of data, and one‐way or two‐way analysis of variance were used to analyze more than two groups of data. *p* < .05 was considered statistically significant.

## 3 RESULTS

### 3.1 Changes in intestinal microbiota diversity

**Figure 1:**
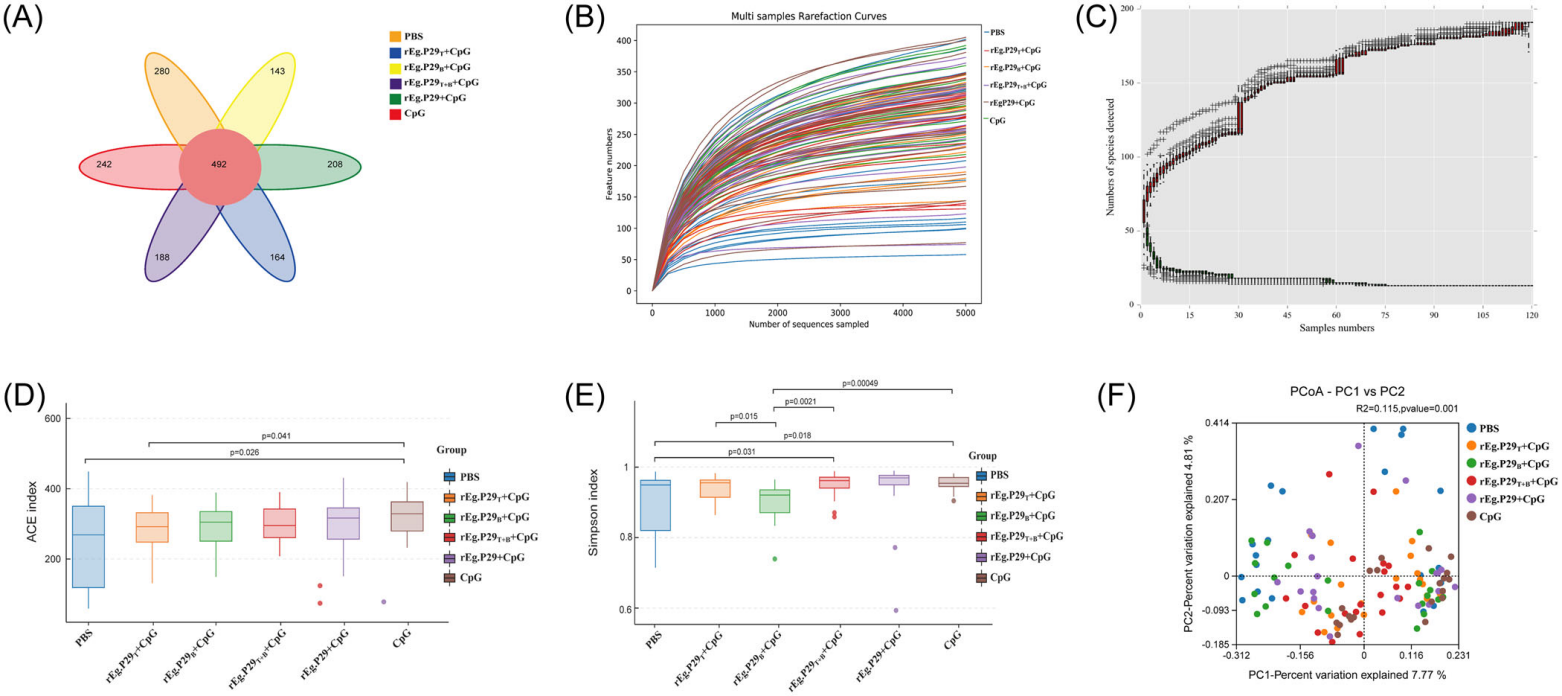
Changes in intestinal microbiota diversity of mice after immunization with different epitope peptide vaccines. (A) Operational taxonomic unit (OTU) clustering Venn diagram of the intestinal microbiota of each group of mice. The petal diagram is a representation of the Venn diagram. The numbers in the middle represent the number of features common to all samples, and the numbers on the petals represent the number of features specific to the samples. (B) Dilution curves for each group of samples, used to verify whether the amount of sequencing data is sufficient to reflect the species diversity in the samples and indirectly to observe the richness of the species in the samples. A gradual rising and flattening of the curve indicates a reasonable amount of data for subsequent analysis. (C) A cumulative species curve of the samples reflecting the relationship between the number of samples and number of species annotated. As the curve flattens, the common species in this environment becomes saturated, and the data can be further analyzed. (D, E) ACE and Simpson indices to indicate species abundance and species diversity, respectively. (F) Principal coordinates analysis (PCoA) was used to represent the differences in species diversity between six groups of samples, with the dots representing each sample and different colors representing the different groups. The horizontal and vertical coordinates represent the two characteristics that are the most different between the samples, with the main effect indicated in the form of a percentage. ACE, abundance‐based coverage estimator.

We tested 120 fecal samples and finally obtained 1717 OTUs (Operational Taxonomic Unit), including 492 common OTUs, with 280 specific to the PBS group, 164 specific to the rEg.P29_T_ + CpG group, 143 specific to the rEg.P29_B_ + CpG group, 188 specific to the rEg.P29_T + B_ + CpG group, 208 specific to the rEg.P29 + CpG group, and 242 specific to the CPG group (Figure 1A). The abundance of species in each sample was assessed by plotting a sample rarefaction curve. A rarefaction curve is produced by randomly selecting a certain number of sequences from a sample, counting the number of species represented by these sequences, and constructing a curve in terms of the number of sequences versus the number of species. It is used to validate whether the amount of sequencing data is sufficient to reflect the diversity of species in the sample and indirectly reflect the abundance of each species in the sample. The gradual rising and levelling off of the curve in this study indicated a reasonable amount of sample data for subsequent analyses (Figure 1B). Furthermore, species count curves reflect the number of species sequenced in each sample. In this study, these curves also rose gradually and levelled off at the end, demonstrating that the number of species in the samples can support further experiments (Figure 1C). Alpha diversity is an important measured of microbial diversity that focuses on the degree of species diversity in a locally homogeneous habitat, examining both the richness of species and evenness of the distribution of individuals in the community in a given sample or multiple samples. Typically, indices used to express alpha diversity include the Chao1, abundance‐based coverage estimator (ACE), Shannon, and Simpson indices, among others. The Chao1 and ACE indices measure the abundance of species, while the Shannon and Simpson indices measure the diversity of species. Here, the ACE and Simpson indices were used to evaluate the abundance and diversity of the mouse intestinal flora, and Good's coverage value was used to indicate the sequencing depth of the samples. The value of the six groups of Good's coverage values was 1, indicating that the depth of sequencing met the follow‐up experimental requirements (Table 1). The ACE index was used to indicate the number of species in the sample, with larger ACE indices indicating a greater number of species. In this sample, the ACE index of the rEg.P29_T_ + CpG group was significantly lower than that of the CpG group, with little difference found between the remaining groups (Figure 1D). Additionally, the Simpson index was used to indicate the species diversity, with greater Simpson indices indicating less community diversity. Resultantly, the Community Diversity of the rEg.P29_T + B_ + CpG group was significantly lower than that of the PBS group, and that of the rEg.P29_B_ + CpG group was significantly higher than that of the CpG group (Figure 1E). Beta diversity analysis was used to compare the microbial community composition of different samples as well as between groups of samples. In this study, differences in the structure of the six groups of samples were investigated using principal coordinate analysis. The percentage of the horizontal coordinate was 7.77% and that of the vertical coordinate was 4.81%, suggesting significant differences in the structure of the microbial community in each group of samples (Figure 1F).

**Table 1:** Alpha diversity comparison.

Group	ACE	Simpson	Good's coverage
PBS	251.80 ± 119.65	0.90 ± 0.09	1
rEg.P29_T_ + CpG	279.40 ± 71.53	0.94 ± 0.04	1
rEg.P29_B_ + CpG	287.65 ± 66.67	0.90 ± 0.05	1
rEg.P29_T + B_ + CpG	288.75 ± 78.83	0.95 ± 0.04	1
rEg.P29+CpG	294.60 ± 90.37	0.94 ± 0.09	1
CpG	322.90 ± 53.57	0.95 ± 0.02	1

*Note*: ACE and Simpson indices were used to evaluate the abundance and diversity of the mouse intestinal flora, and Good's Coverage values were used to indicate the sequencing depth of the samples.

Abbreviation: ACE, abundance‐based coverage estimator; PBS, phosphate buffer solution.

### 3.2 Species annotation results

**Figure 2:**
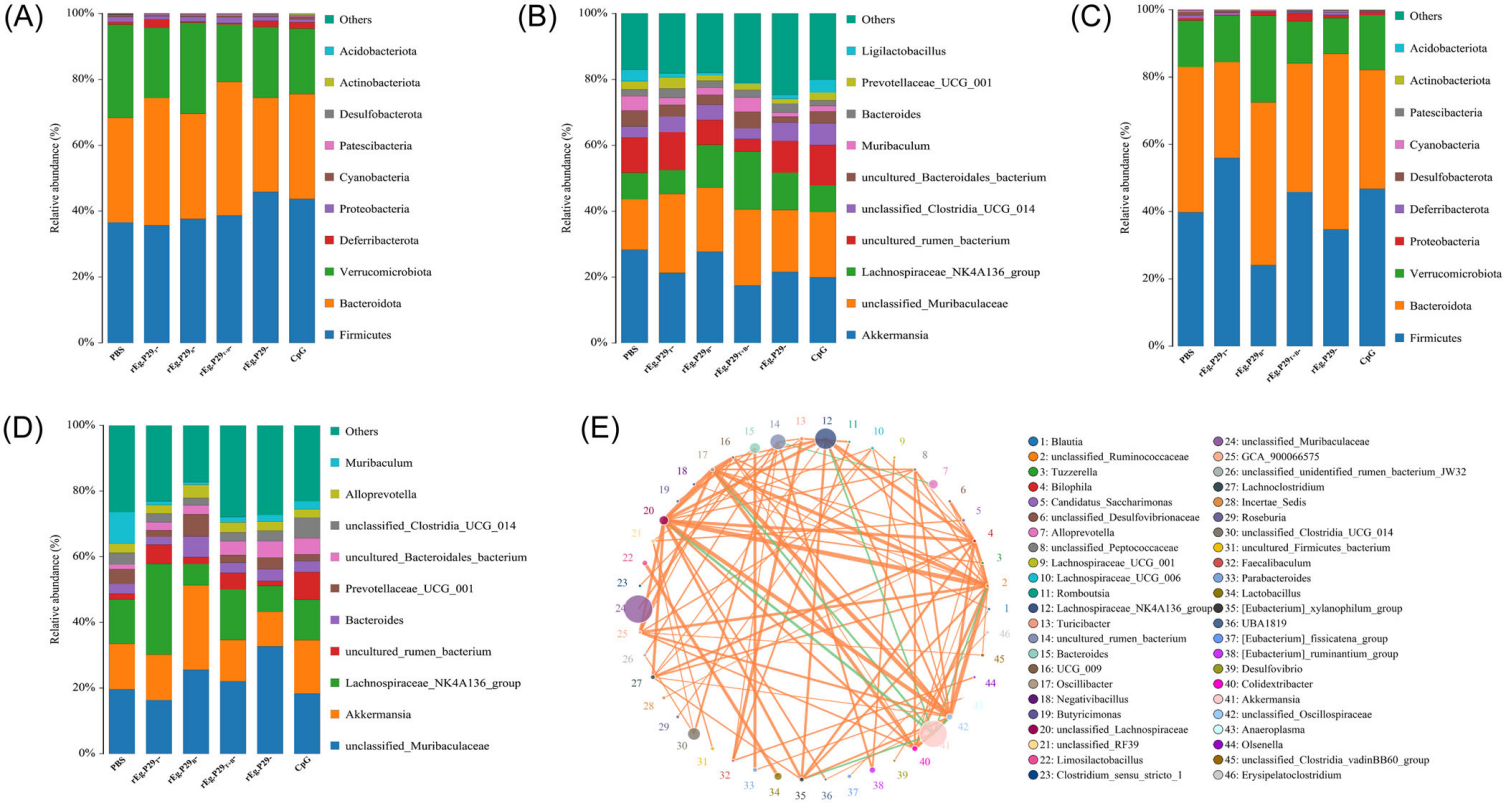
Phylum‐ and genus‐level annotations of samples from each group at 2 and 16 weeks after immunization. Histograms of the species distributions at the (A) phylum level and (B) genus level 2 weeks after booster immunization. Histogram of species distribution at the (C) phylum level and (D) genus level 16 weeks after booster immunization. (E) Network correlation maps drawn by taking the top 50 genera in this study. Network mapping is a form of correlation analysis, using the abundance of each species in each sample, changes in the Spearman rank correlation analysis, and filtering for correlations > 0.1 and *p* Values < .05 to construct a correlation network. (The length of the color block indicates the proportion of relative abundance occupied by the species, the circle represents the species, the size of the circle represents the average abundance size of the species, the line represents the correlation between the two species, the thickness of the line represents the strength of the correlation, and the color of the line represents a positive [orange] or negative correlation [green]).

Based on the clustering of OTUs, we analyzed the microbial taxon composition of each group of samples at the phylum and genus levels. The results highlighted that a total of 15 taxa were detected at the phylum level at the two postimmunisation time points (Weeks 2 and 16), and histograms of relative abundance were plotted for the species ranked in the top 10 most abundant. Although Firmicutes, Bacteroidota, and Verrucomimicrobiota were the dominant phylum in all groups, their relative proportions varied (Figure 2A,C). A total of 192 taxa were detected at the genus level, with Akkermansia, unclassified_Muribaculaceae, Lachnospiraceae_NK4A136_group, and uncultured_rumen_bacterium found to be the dominant genera (Figure 2B,D). To verify the accuracy of the results, we took the top 50 genera and drew a correlation network diagram, which indicated consistent results with those obtained from the histogram (Figure 2E).

### 3.3 Analysis of differences between groups

To further investigate the differences between the gut microbiota of mice immunized with each of the three peptide vaccines and the control group, we analyzed the species abundance data between the groups by t‐test using Metastats software to obtain *p* Values. The species leading to differences in the composition of the samples of the two groups were filtered according to their *p* Values. As there were too many subgroups to analyze all of them in this study, we performed intergroup significance analyses at the species level and presented the top 10 most significant groups of data in a table. Our results showed that Lactobacillus_taiwanensis increased significantly (*p* < .05) in all four immunized groups compared to those of the control group 2 weeks after immunization, while Ruminococcus_bromii, Turicimonas_muris, and Parabacteroides_ merdae all decreased (*p* < .05). At 16 weeks post‐immunization, Muribaculum_intestinale, Desulfovibrio_fairfieldensis, Turicibacter_sanguinis, Clostridiales_bacterium_CIEAF_020, unclassified_Allobaculum, and uncultured_Clostridiales_bacterium in the four immunized groups, had significantly decreased (*p* < .05) compared to those in the control group. In addition to the flora that were consistently raised or lowered in all immunized groups compared to the control group, we screened for significantly different flora specific to each group. Among the bacteria that differed between the rEg.P29_T_ + CpG group and control group, Bacteroides_acidifaciens, Bilophila_wadsworthia, unclassified_Alistipes, unclassified_[Clostridium]_ methylpentosum_group, and so forth, were significantly increased, while Turicibacter_‐_sanguinis, Clostridiales_bacterium_CIEAF_020, Bacteroides_stercorirosoris, unclassified_Incertae_Sedis, and so forth, were significantly decreased (Tables 2 and 3 and Supporting Information S1: Tables 1 and 5). Moreover, the flora of the rEg.P29_B_ + CpG group was significantly different from that of the control group, with a significant increase in unclassified_Alistipes, unclassified_Prevotellaceae_UCG_001, Lactobacillus_intestinalis, Clostridium_disporicum, and so forth, and decrease in Clostridiales_bacterium_CIEAF_020, unclassified_[Eubacterium]_xylanophilum_group, uncultured_Ruminococcaceae_bacterium, Ruminococcus_flavefaciens, and so forth, (Tables 4 and 5 and Supporting Information S1: Tables 2 and 6). The flora of the rEg.P29_T + B_ + CpG group was significantly different from that of the control group as well, with a significant increase in Bacteroides_caecimuris, Firmicutes_bacterium_M10_2, unclassified_[Clostridium]_methylpentosum_group, Alistipes_sp_CHKCI003, and so forth, and decrease in uncultured_rumen_bacterium, Bacteroides_thetaiotaomicron, Ligilactobacillus_murinus, unclassified_Clostridia_UCG_014, and so forth, (Tables 6 and 7 and Supporting Information S1: Tables 3 and 7). Finally, the flora of the rEg.P29 + CpG group was significantly different from that of the control group, with a significant increase in unclassified_Alistipes, Ileibacterium_valens, unclassified_NK4A214_group, Lactobacillus_intestinalis, and so forth, and a significant decrease in Muribaculum_intestinale, Trichinella_pseudospiralis, uncultured_Bacteroidales_bacterium, and so forth,(Tables 8 and 9 and Supporting Information S1: Tables 4 and 8).

**Table 2:** Analysis of differences between groups.

Differential flora（2 weeks post‐immunisation）	CpG(Mean)	rEg.P29_T_‐(Mean)	*p* Value	variation trend
Lactobacillus_taiwanensis	0.0010954	0.003935221	.000381	↑
Bacteroides_acidifaciens	0.0041617	0.01718646	.002497	↑
unclassified_[Clostridium]_methylpentosum_group	0.0000195	0.000412728	.005159	↑
Clostridium_sp._Clone_49	0.0002014	0.001744391	.015564	↑
unclassified_[Eubacterium]_ruminantium_group	0.0069269	0.017593097	.01911	↑
Streptococcus_danieliae	0	0.000198765	.023342	↑
Phocaeicola_dorei	0.0000736	0.000629644	.025748	↑
Ruminococcus_flavefaciens	0.000795	0.00096054	.034294	↑
unclassified_[Eubacterium]_siraeum_group	0.0006318	0.010941271	.04125	↑
unclassified_Ruminococcus	0.0000963	0.000798579	.045155	↑
Lactobacillus_johnsonii	0.0149804	0.000213278	.000157	↓
Parabacteroides_merdae	0.0014423	0.0000216	.000157	↓
Turicibacter_sanguinis	0.0062266	0.001444044	.000157	↓
Turicimonas_muris	0.0050792	0.000182837	.000157	↓
Lactobacillus_intestinalis	0.0016082	0.000278587	.000507	↓
Bacteroides_stercorirosoris	0.0037512	0.000886574	.001152	↓
unclassified_Clostridia	0.0005943	0.000115303	.002827	↓
unclassified_Incertae_Sedis	0.0008069	0.0000512	.005159	↓
unclassified_RF39	0.0062467	0.002358189	.005159	↓
Lachnospiraceae_bacterium_10_1	0.0073127	0	.008151	↓

**Table 3:** Analysis of differences between groups.

Differential flora (16 weeks post‐immunisation)	CpG (mean)	rEg.P29_T_‐(mean)	*p* Value	Variation trend
Bilophila_wadsworthia	0.000848	0.004316	.001152	↑
unclassified_Lachnospiraceae_NK4A136_group	0.062443	0.179295	.001499	↑
unclassified_[Eubacterium]_siraeum_group	0.000173	0.002244	.001499	↑
unclassified_Roseburia	0.000332	0.003178	.003197	↑
Clostridiales_bacterium_CIEAF_021	0.001068	0.00773	.005159	↑
unclassified_Oscillospiraceae	0.003504	0.015803	.005159	↑
Clostridium_sp._Culture_Jar_44	0.000115	0.001688	.008151	↑
Lactobacillus_reuteri	0.001498	0.007047	.008151	↑
Acutalibacter_muris	7.98E‐05	0.0004	.04125	↑
Clostridiales_bacterium_CIEAF_020	0.010833	3.74E‐05	.000157	↓
unclassified_Allobaculum	0.015442	0	.000157	↓
Turicibacter_sanguinis	0.004726	0.000147	.000507	↓
uncultured_Clostridiales_bacterium	0.016513	0.003434	.00067	↓
uncultured_Ruminococcaceae_bacterium	0.001768	0.000326	.00067	↓
unclassified_Erysipelotrichaceae	0.004741	0.001098	.001152	↓
unclassified_unidentified_rumen_bacterium_JW32	0.002844	0.000323	.002827	↓
unclassified_[Eubacterium]_fissicatena_group	0.001912	0.000144	.003611	↓
Parabacteroides_goldsteinii	0.000833	7.44E‐05	.005159	↓

**Table 4:** Analysis of differences between groups.

Differential flora (2 weeks post‐immunisation)	CpG (mean)	rEg.P29_B_‐ (mean)	*p* Value	Variation trend
Lactobacillus_taiwanensis	0.0010954	0.009424449	.000157	↑
Trichinella_pseudospiralis	0.0008765	0.042150009	.000157	↑
Parasutterella_excrementihominis	0.002358	0.009838698	.000285	↑
Lachnospiraceae_bacterium_M18_1	0.0004606	0.002098237	.002497	↑
Phocaeicola_dorei	0.0000736	0.000521204	.004586	↑
unclassified_[Eubacterium]_siraeum_group	0.0006318	0.00243985	.005159	↑
unclassified_[Eubacterium]_coprostanoligenes_group	0.0020553	0.004177272	.006502	↑
Romboutsia_ilealis	0.0004126	0.00103245	.008151	↑
Bifidobacterium_pseudolongum	0.0000844	0.000530841	.012611	↑
Clostridium_sp._Clone_49	0.0002014	0.001307738	.015564	↑
Parabacteroides_merdae	0.0014423	0.0000782	.000157	↓
uncultured_Ruminococcaceae_bacterium	0.0026912	0.000344872	.000157	↓
Lachnospiraceae_bacterium_G11	0.0042705	0.000109078	.000381	↓
Lactobacillus_johnsonii	0.0149804	0.003323215	.000381	↓
uncultured_rumen_bacterium	0.1228007	0.075663565	.000881	↓
Clostridiales_bacterium_CIEAF_020	0.0192332	0.000251652	.002497	↓
Clostridium_sp._SN17	0.0020585	0	.008151	↓
unclassified_Defluviitaleaceae_UCG_011	0.0004581	0.0000151	.01133	↓
Ligilactobacillus_murinus	0.0381283	0.007540234	.015564	↓
Turicimonas_muris	0.0050792	0.002678196	.015564	↓

**Table 5:** Analysis of differences between groups.

Differential flora (16 weeks post‐immunisation)	CpG (mean)	rEg.P29_B_‐ (mean)	*p* Value	Variation trend
Lactobacillus_intestinalis	0.0002037	0.0042634	.000157	↑
Turicimonas_muris	0.0005603	0.0051269	.000157	↑
unclassified_Prevotellaceae_UCG_001	0.0197634	0.0683829	.000157	↑
Alistipes_sp._CHKCI003	0.0015251	0.0058866	.000285	↑
Bacteroides_caecimuris	0.0078171	0.0239573	.000881	↑
Lachnospiraceae	0.0013074	0.0152832	.00194	↑
unclassified_Clostridia_vadinBB60_group	0.0010659	0.0053814	.003197	↑
Limosilactobacillus_reuteri	0.002645	0.0076627	.006502	↑
unclassified_Muribaculaceae	0.1836835	0.2550202	.010165	↑
Lactobacillus_reuteri	0.0014982	0.0046192	.012611	↑
Clostridiales_bacterium_CIEAF_020	0.0108325	0	.000157	↓
unclassified_Allobaculum	0.0154417	0	.000157	↓
unclassified_[Eubacterium]_xylanophilum_group	0.0077775	0.0004334	.000507	↓
Turicibacter_sanguinis	0.0047264	0.0003633	.00067	↓
unclassified_unidentified_rumen_bacterium_JW32	0.0028443	0	.00067	↓
unclassified_[Eubacterium]_ruminantium_group	0.0035545	2.10E‐05	.000769	↓
uncultured_Clostridiales_bacterium	0.0165134	0.0034363	.001152	↓
uncultured_Ruminococcaceae_bacterium	0.0017683	0.0005272	.00194	↓
Ruminococcus_champanellensis	0.0004738	0	.002497	↓
uncultured_rumen_bacterium	0.0853087	0.020095	.003197	↓

### 3.4 Picrust2 functional prediction

**Figure 3:**
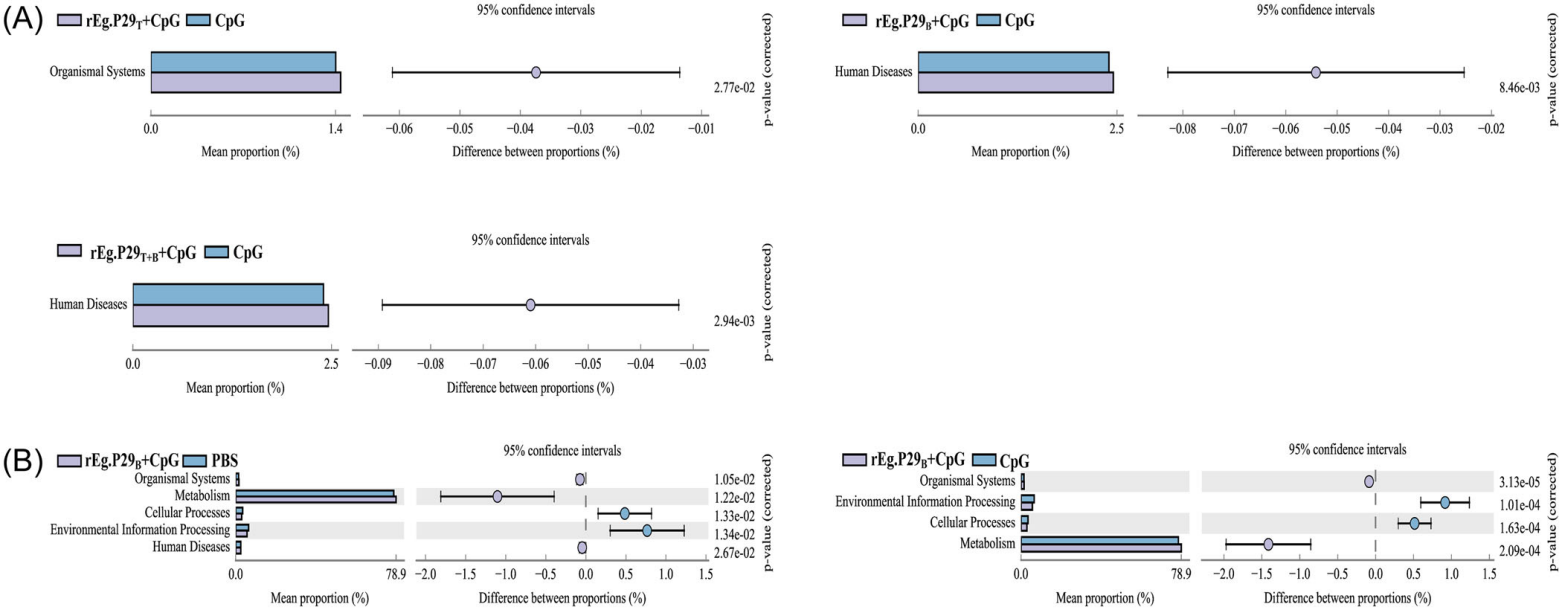
Differences in enriched KEGG metabolic pathways of immunized groups compared to those of controls. (A) Differential metabolic pathways between the rEg.P29_T_ + CpG, rEg.P29_B_ + CpG, and rEg.P29_T + B_ + CpG groups and control group 2 weeks after booster immunization. (B) Differential metabolic pathways between the rEg.P29_B_ + CpG group and control group 16 weeks after immunization. (The proportions of abundance of different functions in the two sets of samples are shown on the left in the image, the proportions of differences in functional abundance within the 95% confidence interval are shown in the middle, and the rightmost value is the *p* Value.).

Through analysis of the KEGG metabolic pathways, any differences and changes in the functional genes of the microbial communities between samples groups were observed. This is an effective means of studying changes in metabolic function in response to environmental changes. Picrust2 software was used to compare the species composition information obtained from the 16 S rRNA sequencing data, revealing that the differential flora was enriched in multiple KEGG metabolic pathways. Specifically, when comparing the rEg.P29_T_ + CpG, rEg.P29_B_ + CpG, and rEg.P29_T + B_ + CpG groups to the CpG group, the following metabolic pathways were enriched at 2 weeks after booster immunization: Organismal Systems, Human Diseases, respectively (Figure 3A). Additionally, the following metabolic pathways were found to be enriched in the rEg.P29_B_ + CpG group compared to the control group at 16 weeks after booster immunization: Organismal Systems, Metabolism, Cellular Processes, Environmental Information Processing, and Human Diseases (Figure 3B).

**Table 6:** Analysis of differences between groups.

Differential flora (2 weeks post‐immunisation)	CpG (mean)	rEg.P29_T + B_‐ (mean)	*p* Value	Variation trend
Lactobacillus_taiwanensis	0.0010954	0.009634152	.000157	↑
unclassified_Allobaculum	0	0.03221742	.000157	↑
Parasutterella_excrementihominis	0.002358	0.015328126	.000381	↑
Muribaculum_intestinale	0.0169535	0.045103641	.000507	↑
Bacteroides_caecimuris	0.0008373	0.003807736	.000881	↑
Alistipes_sp._CHKCI003	0.0015789	0.005132551	.001499	↑
unclassified_[Clostridium]_methylpentosum_group	0.0000195	0.000840994	.003197	↑
unclassified_Lachnospiraceae_NK4A136_group	0.0425171	0.108924623	.010165	↑
unclassified_Colidextribacter	0.0034402	0.006048473	.012611	↑
uncultured_Bacteroidales_bacterium	0.0421451	0.068644559	.01911	↑
Lactobacillus_johnsonii	0.0149804	0.000466151	.000157	↓
uncultured_rumen_bacterium	0.1228007	0.038915893	.000157	↓
Parabacteroides_merdae	0.0014423	0.0000606	.000212	↓
Turicimonas_muris	0.0050792	0.000313621	.000212	↓
Clostridium_disporicum	0.0015064	0	.00067	↓
Christensenella_minuta	0.0008323	0.000143846	.000881	↓
Ligilactobacillus_murinus	0.0381283	0.004720764	.001499	↓
unclassified_Clostridia_UCG_014	0.0647543	0.030828315	.001499	↓
unclassified_Clostridia	0.0005943	0.000112667	.001706	↓
Turicibacter_sanguinis	0.0062266	0.003185625	.00194	↓

**Table 7:** Analysis of differences between groups.

Differential flora（16 weeks post‐immunisation）	CpG (mean)	rEg.P29_T + B_‐(mean)	*p* Value	Variation trend
Firmicutes_bacterium_M10_2	1.44E‐05	0.0109141	.000157	↑
unclassified_Dubosiella	0	0.0255143	.00067	↑
Lachnospiraceae_bacterium_M18_1	0.0004875	0.0017322	.021134	↑
unclassified_[Eubacterium]_nodatum_group	0.0007283	0.0014109	.031209	↑
Lactobacillus_intestinalis	0.0002037	0.0010992	.04125	↑
unclassified_Marvinbryantia	0.0002116	0.0010353	.045155	↑
Acutalibacter_muris	7.98E‐05	0.0004035	.049366	↑
Clostridiales_bacterium_CIEAF_020	0.0108325	0.0002161	.000157	↓
unclassified_Allobaculum	0.0154417	0	.000157	↓
Bacteroides_thetaiotaomicron	0.0005485	7.29E‐05	.004586	↓
Parabacteroides_merdae	0.0007498	0.0001206	.012611	↓
uncultured_Ruminococcaceae_bacterium	0.0017683	0.0007913	.015564	↓
Clostridiales_bacterium_CIEAF_013	0.0012051	0.0001243	.023342	↓
unclassified_Clostridia_UCG_014	0.0637516	0.0253473	.028366	↓
uncultured_Clostridiales_bacterium	0.0165134	0.0095371	.028366	↓
Turicibacter_sanguinis	0.0047264	0.0011317	.034294	↓
unclassified_Incertae_Sedis	0.0009323	1.92E‐05	.04125	↓

**Table 8:** Analysis of differences between groups.

Differential flora (2 weeks post‐immunisation)	CpG (mean)	rEg.P29 ‐ (mean)	*p* Value	Variation trend
Lactobacillus_taiwanensis	0.0010954	0.006362233	.000285	↑
rumen_bacterium_NK4A214	0.0001826	0.001209507	.000285	↑
Bacteroides_acidifaciens	0.0041617	0.013260249	.001152	↑
unclassified_[Eubacterium]_ruminantium_group	0.0069269	0.041566884	.004586	↑
Parasutterella_excrementihominis	0.002358	0.007124508	.005159	↑
Ruminococcus_flavefaciens	0.000795	0.001249256	.034294	↑
butyrate_producing_bacterium_L2_12	0.0001495	0.000529478	.049366	↑
unclassified_Family_XIII_AD3011_group	0.0005529	0.001364703	.049366	↑
unclassified_[Eubacterium]_coprostanoligenes_group	0.0020553	0.003810905	.049366	↑
Lactobacillus_johnsonii	0.0149804	0.001940723	.000157	↓
Parabacteroides_merdae	0.0014423	0.000014	.000157	↓
Turicimonas_muris	0.0050792	0.00044021	.000157	↓

**Table 9:** Analysis of differences between groups.

Differential flora（16 weeks post‐immunisation）	CPG (mean)	rEg.P29‐(mean)	*p* Value	Variation trend
Ileibacterium_valens	8.24E‐05	0.0113125	.000881	↑
unclassified_Muribaculaceae	0.1836835	0.3344427	.00194	↑
Lachnospiraceae_bacterium_DW52	0	0.0020507	.002497	↑
unclassified_Erysipelotrichaceae	0.0047412	0.015444	.003197	↑
Lactobacillus_intestinalis	0.0002037	0.0020299	.003611	↑
unclassified_Erysipelatoclostridium	0.0013037	0.0083178	.005795	↑
unclassified_Colidextribacter	0.0042455	0.0080321	.006502	↑
Clostridiales_bacterium_CIEAF_019	8.14E‐05	0.0007341	.028366	↑
Phocaeicola_vulgatus	0.0006257	0.0026703	.034294	↑
Clostridiales_bacterium_CIEAF_026	0.0005583	0.0029059	.037635	↑
Clostridiales_bacterium_CIEAF_020	0.0108325	8.19E‐05	.000157	↓
unclassified_Allobaculum	0.0154417	0.0026135	.000285	↓
Turicibacter_sanguinis	0.0047264	0.0001555	.000381	↓
uncultured_rumen_bacterium	0.0853087	0.0150844	.000507	↓
uncultured_Clostridiales_bacterium	0.0165134	0.0049817	.000881	↓
Trichinella_pseudospiralis	0.0400656	0.0067509	.001152	↓
Bacteroides_thetaiotaomicron	0.0005485	0.0001146	.008151	↓
Clostridium_sp._Clone_27	0.0020807	0	.008151	↓
unclassified_unidentified_rumen_bacterium_JW32	0.0028443	0.0004846	.008151	↓
Parabacteroides_merdae	0.0007498	0.0001488	.012611	↓

*Note*: “↑” and “↓” indicate that the intestinal flora is upregulated and downregulated.

“↑” and “↓” indicate that the intestinal flora is upregulated and downregulated.

## 4 DISCUSSION

Over the past 20 years, the global distribution of echinococcosis has not significantly changed. Although a few island nations have announced the elimination of echinococcosis, its incidence and dissemination rates in some areas continue to increase.^33^, ^34^, ^35^ Empirical evidence for the successful treatment of *E. granulosus* and *Echinococcus multilocularis* infections is lacking owing to the rarity of clinical studies. Thus, no significant and widely accepted treatment is currently available. Vaccination is an effective measure to prevent echinococcosis.^36^, ^37^ In recent years, many studies have characterized the protective antigens of *E. granulosus* and their role in the immunization of various animal hosts.^38^ While our previous studies have shown that rEg.P29 can provide more than 90% immunoprotection in both sheep and mice, these studies were limited to exploring the immunogenicity of genetically engineered vaccines.^39^ To better understand the real potential of an rEg.P29 vaccine, we designed three peptide vaccines based on rEg.P29 and verified their immunogenicity.

It has been shown that commensal bacteria, particularly those within the gut microbiota, influence vaccine‐mediated antigen‐specific immune responses and contribute to the induction of nonspecific responses by “training” natural immune cells.^40^ Therefore, it is necessary to consider the interactions between the gut microbiota and immune cells to help ensure optimal immunogenicity of vaccines and adjuvants. With the continuous progress of PCR and nucleic acid research technologies, 16 S rRNA gene detection has emerged as the most widely used tool for bacterial identification and detection.^41^ To date, there have been numerous studies on the relationship between parasitic infections and intestinal flora. One of these studies found that *Eisenbergiella* and *Parabacteroides spp*. increased significantly when mice were infected with *E. granulosus*, and that their increase was likely to have a serious impact on the health of the organism.^32^ Although vaccination, an effective preventive measure against CE, is likely to reduce harmful bacteria, increase the abundance of beneficial bacteria, and thus improve the overall body condition, little research has been reported to this regard so far. Therefore, in the present study, we sequenced the 16 S rRNA genes of the intestinal flora of mice immunized with three rEg.P29‐based peptide vaccines to provide a better theoretical basis for the study of vaccines against CE.

Early studies in germ‐free mice showed that bacterial colonization is necessary for normal gut‐associated lymphoid tissue (GALT) development and that specific microbiota can increase IgA secretion.^42^ Oh et al.^43^ also found that gut bacteria can be used as adjuvants for vaccination, as evidenced by the ability of flagellated bacteria to restore the antibody response to influenza after both antibiotic treatment and influenza vaccination. Our findings revealed a significant increase in Lactobacillus_taiwanensis and decrease in Ruminococcus bromii in all four immunized groups compared to those of the control group at the species level 2 weeks after immunization. In 2006, Lactobacillus_taiwanensis was isolated from silage cattle feed in Taiwan, and after a series of characterizations, it was found to phylogenetically belong to the genus *Lactobacillus*.^44^ As a highly safe microorganism, *Lactobacillus* not only maintains the balance of the body's microbiological system, but also helps the body to lower blood lipids and blood pressure as well as lower the risk of cancer. One of the studies found that *Lactobacillus* could alleviate diarrhoea caused by enterotoxin‐producing *E. coli* (ETEC) in mice.^45^ Ruminococcus_bromii is an prominent member of the Firmicutes phylum. While some studies indicate a positive association between the genus *Ruminococcus* and type II diabetes mellitus (T2D), others have come to the opposite conclusion, with an increase in the abundance of Ruminococcus bromii following bariatric surgery and diabetes remission.^46^ Furthermore, a particularly significant association between Crohn's disease and *Ruminococci* was found by Henke et al.^47^ Nevertheless, studies on Ruminococcus_bromii in relation to parasitic infections have not yet been reported. The rEg.P29_T_ + CpG group showed a significant increase in unclassified_Alistipes, Bacteroides_acidifaciens, and so forth, compared to those of the control group. Unclassified_Alistipes and Bacteroides_acidifaciens all belong to the Bacteroidetes phylum, which is abundant in the bacterial communities of healthy organisms and promotes recruitment, maturation of immune cells, and modulation of the immune response.^48^ Wang et al.^49^ found that *Bacteroides acidifaciens* in the gut was protective against CD95‐mediated liver injury. Indeed, the main site of infection for echinococcosis is the liver, apparently in line with our expectations. Furthermore, Alistipes are producers of SCFA, which are not only used as nutrients to provide energy, but also have important physiological regulatory roles, such as regulating cell proliferation and differentiation, apoptosis, immune response, nutrient uptake, and lipid metabolism.^50^ Lachnospiraceae_bacterium_M18_1 was significantly elevated in the rEg.P29_B_ + CpG group of mice compared to that of the control group, and Clostridiales_bacterium_CIEAF_020 and Clostridium_sp._SN17 were significantly decreased. Lachnospiraceae_bacterium_M18_1 belongs to the Firmicutes, a phylum present in the intestinal tract of most healthy individuals, and may be a potentially beneficial bacterium involved in the metabolism of a variety of carbohydrates. For example, it plays a role in the fermentation of pectin, a complex dietary fiber and prebiotic in fruits and vegetables, leading to the production of acetic and butyric acids and providing a main source of energy for the host. Hamilton et al.^51^ found that genera associated with maintenance of remission were predominantly enriched in the Lachnospiraceae family by studying the intestinal flora associated with postoperative relapse in patients with Crohn's disease. Clostridium_sp was identified as an anaerobic, Gram‐positive, rod‐shaped strain, and it was determined that the strain belongs to the genus *Clostridium*. While Cerniglia et al. found that Clostridium is a harmful bacterial genus, the present study of harmful *Clostridium* is still in its early stages. *Clostridium difficile*, *Clostridium perfringens*, and *Clostridium botulinum* are the three main harmful Clostridium species that have been extensively studied, while the pathogenesis of other species of harmful Clostridium species is less well research, requiring further in‐depth study to provid einsight for the prevention and treatment of various diseases.^52^, ^53^, ^54^ Within the rEg.P29_T + B_ + CpG group, unclassified_Candidatus_Saccharimonas, unclassified_Muribaculaceae, and unclassified_Lachnospiraceae_NK4A136_group were significantly elevated compared to those of the control group. A previous study suggested that Candidatus_Saccharimonas may be an important factor in immune recovery in immunodeficient patients and could be used in the future as a biomarker for screening immune responses in HIV‐infected patients.^55^ Muribaculaceae, formally known as a member of the S24‐7 family, is highly abundant in the mouse intestine and belongs to the Bacteroidetes. Although changes in Muribaculaceae are primarily associated with various dietary treatments, host conditions, or rodent colonization processes, the specific functions of the family have not yet been thoroughly invesigated.^56^ Li et al. ^57^ found that Lachnospiraceae_NK4A136_group exhibited anti‐inflammatory properties, promoted the repair of intestinal mucosa, and effectively alleviated colitis in a mouse model of colitis. Decreases in Turicibacter_sanguinis, Clostridiales_bacterium_CIEAF_020, unclassified_Allobaculum, Muribaculum_intestinale, and Desulfovibrio_fairfieldensis were found at the species level at 16 weeks post‐immunization compared to those of the control group. While the original strain of *Turicibacter sanguinis* was isolated from a blood culture of a febrile patient with acute appendicitis, some *Turicibacter* bacteria have been reported to have a pathogenic lifestyle and are commonly associated with host inflammatory responses.^58^ This may be attributed to the fact that vaccine immunization triggers a strong immune response in mice, leading to a decline in some of the flora that have been implicated in the inflammatory response. Desulfovibrio_fairfieldensis is an opportunistic pathogen that is widespread in the human gut and can cause serious infectious diseases.^59^ Interestingly, no detectable changes in Lactobacillus_taiwanensis were seen in any of the four immunized groups at 16 weeks compared to 2 weeks post‐immunization, while two probiotics, Lactobacillus_reuteri and Lactobacillus_intestinalis, increased significantly during that time. Meanwhile, the unclassified_Lachnospiraceae_NK4A136_group bacteria in the rEg.P29_T_ + CpG group were significantly elevated, which was highly consistent with the results of previous studies. There was a significant increase in unclassified_Prevotellaceae_UCG_001 in the rEg.P29_B_ + CpG group. The Prevotellaaceae_UCG___001 bacterium is widely recognized as a probiotic with the ability to promote the production of SCFA.^60^ This is in accordance with the results obtained 2 weeks after immunization. The uncultured_Bacteroidales_bacterium and uncultured_rumen_bacterium of the rEg.P29_T + B_ + CpG group were elevated compared to those of the control group, while the unclassified_Clostridia_UCG_014 was significantly reduced. Zhong et al.^61^ found that enrichment of *Lactobacillus johnsonii* caused remodeling of the gut microbiota by increasing the abundance of Muribaculaceae and *Lactobacillus* and decreasing that of Clostridia UCG_014. The specific mechanisms that lead to changes in intestinal flora after vaccine immunization were explored by means of functional prediction of these differential flora. We found that multiple KEGG metabolic pathways were enriched, namely biological systems, human diseases, metabolism, cellular processes, and environmental information processing. Their specific effects within these pathways remain unknown at this point.

In summary, this study revealed that some probiotics in the intestinal tract of peptide vaccine‐immunized mice were significantly increased compared with those of the control group, such as Lactobacillus_taiwanensis, Alistipes, Lachnospiraceae_NK4A136_group, and Bacteroides_acidifaciens, among others. Moreover, certain harmful or conditionally pathogenic bacteria were also reduced, most which are associated with inflammatory or infectious diseases. This may be related not only to the fact that immunization with rEg.P29_B_ and rEg.P29_T + B_ epitope peptide vaccines can trigger a strong antibody response in mice, but also to the fact that immunization with rEg.P29_T_, rEg.P29_T + B_, or rEg.P29 induces the release of a substantial amount of Th1‐type cytokines. However, there are many deficiencies in this study, the most crucial of which is that an animal infection model was not established to further evaluate the influence of the three peptide vaccines on the intestinal microbiota. To provide more valuable information for the development of a vaccine against CE, we will conduct more in‐depth studies in the future.

## AUTHOR CONTRIBUTIONS

Mingxing Zhu and Wei Zhao were responsible for the design of the experiments and the revision of the thesis; Tingting Zhang and Yongxue Lv were responsible for the construction of animal models, data analysis, and writing of the thesis; Yinqi Zhao and Jihui Yang were responsible for experimental supervision; and Bingshuo Qian and Yazhou Zhu were responsible for the collection of materials.

## ETHICS STATEMENT

All procedures involving experimental animals were reviewed and approved by the ethics committee of Ningxia Medical University and conducted in accordance with institutional and national guidelines on experimental animals. All data acquisition and article writing met ethical and moral requirements.

## Supporting information

Supporting information.

## Data Availability

The data used in this study are presented in the text of the article as well as in the Supporting Information Material and any queries should be directed to the corresponding author.
