# Supplementary material for: Changes in intestinal flora of mice induced by rEg.P29 epitope peptide vaccines
Source: Immun Inflamm Dis. 2023 Nov 22;11(11):e1082. doi: 10.1002/iid3.1082 (PMC10664826; doi:10.1002/iid3.1082)
Supplement: Supplementary file 1 — Supporting information. [file IID3-11-e1082-s001.docx]

**supplementary material**

**supplementary Table 1-8. Analysis of differences between groups**

**Supplementary Table 1**

| Differential flora | PBS(Mean) | rEg.P29_T_-(Mean) | *P* value | variation trend |
| --- | --- | --- | --- | --- |
| unclassified_Alistipes | 0.0000405 | 0.001526435 | 0.000285 | ↑ |
| unclassified_[Clostridium]_methylpentosum_group | 0 | 0.000412728 | 0.002497 | ↑ |
| unclassified_Muribaculaceae | 0.1515185 | 0.240795027 | 0.010165 | ↑ |
| Bacteroides_acidifaciens | 0.0066134 | 0.01718646 | 0.012611 | ↑ |
| Lactobacillus_taiwanensis | 0.0018357 | 0.003935221 | 0.012611 | ↑ |
| unclassified_Candidatus_Saccharimonas | 0.0002981 | 0.001767008 | 0.014019 | ↑ |
| Clostridium_disporicum | 0.0003693 | 0.001438175 | 0.015564 | ↑ |
| Clostridium_sp._Clone_49 | 0.0002623 | 0.001744391 | 0.01911 | ↑ |
| Lachnospiraceae_bacterium_G11 | 0.0004564 | 0.006055644 | 0.023342 | ↑ |
| unclassified_Oscillospiraceae | 0.0009144 | 0.004029171 | 0.023342 | ↑ |
| Ruminococcus_bromii | 0.0101443 | 0 | 0.00067 | ↓ |
| unclassified_Incertae_Sedis | 0.0009518 | 0.0000512 | 0.000881 | ↓ |
| Lactobacillus_johnsonii | 0.0035759 | 0.000213278 | 0.001499 | ↓ |
| Muribaculum_intestinale | 0.0470297 | 0.02155618 | 0.00194 | ↓ |
| Turicibacter_sanguinis | 0.0046775 | 0.001444044 | 0.005159 | ↓ |
| Alistipes_putredinis | 0.0012629 | 0.000200974 | 0.015564 | ↓ |
| Bacteroides_stercorirosoris | 0.0051654 | 0.000886574 | 0.023342 | ↓ |
| Faecalibaculum_rodentium | 0.0018566 | 0.00056662 | 0.025748 | ↓ |

**Supplementary Table 2**

| Differential flora | PBS(Mean) | rEg.P29_B_-(Mean) | *P* value | variation trend |
| --- | --- | --- | --- | --- |
| Turicimonas_muris | 0.0001152 | 0.002678196 | 0.000157 | ↑ |
| unclassified_Alistipes | 0.0000405 | 0.001149728 | 0.000157 | ↑ |
| unclassified_Candidatus_Saccharimonas | 0.0002981 | 0.003242394 | 0.000212 | ↑ |
| Lactobacillus_intestinalis | 0.0003087 | 0.002273941 | 0.000381 | ↑ |
| Lactobacillus_taiwanensis | 0.0018357 | 0.009424449 | 0.00067 | ↑ |
| Clostridium_disporicum | 0.0003693 | 0.002223403 | 0.001152 | ↑ |
| uncultured_Clostridiales_bacterium | 0.0007462 | 0.004734605 | 0.001499 | ↑ |
| Alistipes_massiliensis | 0.0060614 | 0.011127591 | 0.002497 | ↑ |
| Turicibacter_sanguinis | 0.0046775 | 0.009869688 | 0.002497 | ↑ |
| Bacteroides_faecichinchillae | 0.0005582 | 0.001558385 | 0.000589 | ↑ |
| Muribaculum_intestinale | 0.0470297 | 0.022486053 | 0.000285 | ↓ |
| uncultured_Bacteroidales_bacterium | 0.0611793 | 0.041558485 | 0.001152 | ↓ |
| Clostridiales_bacterium_CIEAF_020 | 0.0058405 | 0.000251652 | 0.003197 | ↓ |
| Ruminococcus_bromii | 0.0101443 | 0.001855138 | 0.004586 | ↓ |
| uncultured_Ruminococcaceae_bacterium | 0.0013379 | 0.000344872 | 0.01133 | ↓ |
| uncultured_rumen_bacterium | 0.1064306 | 0.075663565 | 0.012611 | ↓ |
| unclassified_A2 | 0.0003722 | 0 | 0.023342 | ↓ |
| Ruminococcus_flavefaciens | 0.0013158 | 0.000199793 | 0.037635 | ↓ |
| unclassified_Prevotellaceae_UCG_001 | 0.025494 | 0.016359349 | 0.04125 | ↓ |

**Supplementary Table 3**

| Differential flora | PBS(Mean) | rEg.P29_T+B_- (Mean) | *P* value | variation trend |
| --- | --- | --- | --- | --- |
| unclassified_Allobaculum | 0 | 0.03221742 | 0.000157 | ↑ |
| Lactobacillus_taiwanensis | 0.0018357 | 0.009634152 | 0.000381 | ↑ |
| unclassified_Candidatus_Saccharimonas | 0.0002981 | 0.003759581 | 0.00067 | ↑ |
| unclassified_Ruminococcaceae | 0.0000377 | 0.00097329 | 0.000881 | ↑ |
| unclassified_Muribaculaceae | 0.1515185 | 0.231502613 | 0.001152 | ↑ |
| Bacteroides_caecimuris | 0.0014777 | 0.003807736 | 0.002497 | ↑ |
| unclassified_[Clostridium]_methylpentosum_group | 0 | 0.000840994 | 0.002497 | ↑ |
| unclassified_Clostridia_vadinBB60_group | 0.0004386 | 0.002347009 | 0.004072 | ↑ |
| Alistipes_sp._CHKCI003 | 0.0021791 | 0.005132551 | 0.006502 | ↑ |
| unclassified_Lachnospiraceae_NK4A136_group | 0.0396837 | 0.108924623 | 0.008151 | ↑ |
| uncultured_rumen_bacterium | 0.1064306 | 0.038915893 | 0.000157 | ↓ |
| Ruminococcus_bromii | 0.0101443 | 0 | 0.00067 | ↓ |
| unclassified_Incertae_Sedis | 0.0009518 | 0 | 0.00067 | ↓ |
| Lactobacillus_johnsonii | 0.0035759 | 0.000466151 | 0.003197 | ↓ |
| Ligilactobacillus_murinus | 0.0335765 | 0.004720764 | 0.005159 | ↓ |
| unclassified_[Eubacterium]_ruminantium_group | 0.0163547 | 0.002318193 | 0.008151 | ↓ |
| Faecalibaculum_rodentium | 0.0018566 | 0.000538231 | 0.009108 | ↓ |
| Ruminococcus_flavefaciens | 0.0013158 | 0.0000657 | 0.01911 | ↓ |
| Akkermansia_muciniphila | 0.3115171 | 0.178893946 | 0.049366 | ↓ |

**Supplementary Table 4**

| Differential flora | PBS(Mean) | rEg.P29 - (Mean) | *P* value | variation trend |
| --- | --- | --- | --- | --- |
| rumen_bacterium_NK4A214 | 0.0001408 | 0.001209507 | 0.000157 | ↑ |
| unclassified_Alistipes | 0.0000405 | 0.00189276 | 0.000285 | ↑ |
| unclassified_Candidatus_Saccharimonas | 0.0002981 | 0.00160105 | 0.001499 | ↑ |
| butyrate_producing_bacterium_L2_12 | 0 | 0.000529478 | 0.002497 | ↑ |
| Lactobacillus_taiwanensis | 0.0018357 | 0.006362233 | 0.004072 | ↑ |
| unclassified_Family_XIII_AD3011_group | 0.0002306 | 0.001364703 | 0.008151 | ↑ |
| unclassified_NK4A214_group | 0.0003794 | 0.001067821 | 0.01133 | ↑ |
| Lactobacillus_intestinalis | 0.0003087 | 0.001434689 | 0.012611 | ↑ |
| Clostridium_disporicum | 0.0003693 | 0.001761847 | 0.014019 | ↑ |
| unclassified_Ruminococcaceae | 0.0000377 | 0.001836172 | 0.015564 | ↑ |
| Muribaculum_intestinale | 0.0470297 | 0.012339184 | 0.000157 | ↓ |
| uncultured_Bacteroidales_bacterium | 0.0611793 | 0.028724747 | 0.000157 | ↓ |
| Ruminococcus_bromii | 0.0101443 | 0 | 0.00067 | ↓ |
| unclassified_Prevotellaceae_UCG_001 | 0.025494 | 0.014325973 | 0.015564 | ↓ |

**Supplementary Table 5**

| Differential flora | PBS(Mean) | rEg.P29_T_-(Mean) | *P* value | variation trend |
| --- | --- | --- | --- | --- |
| unclassified_[Eubacterium]_ruminantium_group | 8.14E-05 | 0.007435 | 0.000157 | ↑ |
| unclassified_Alistipes | 0.00098829 | 0.004085 | 0.000285 | ↑ |
| Alistipes_putredinis | 0.00064052 | 0.001668 | 0.005159 | ↑ |
| uncultured_rumen_bacterium | 0.01622958 | 0.047562 | 0.005159 | ↑ |
| Clostridium_sp._Clone_27 | 0 | 0.003686 | 0.008151 | ↑ |
| Lachnospiraceae_bacterium_10_1 | 0.00018242 | 0.006855 | 0.008151 | ↑ |
| Lactobacillus_reuteri | 0.00160352 | 0.007047 | 0.008151 | ↑ |
| unclassified_[Eubacterium]_siraeum_group | 0.00043229 | 0.002244 | 0.010165 | ↑ |
| Clostridium_sp._Culture_Jar_44 | 0.00022964 | 0.001688 | 0.012611 | ↑ |
| unclassified_Lachnospiraceae_NK4A136_group | 0.07199093 | 0.179295 | 0.012611 | ↑ |
| Desulfovibrio_fairfieldensis | 0.00798317 | 0 | 0.000157 | ↓ |
| Muribaculum_intestinale | 0.09567714 | 0.011978 | 0.000157 | ↓ |
| Alistipes_sp._CHKCI003 | 0.00322376 | 0.000797 | 0.003197 | ↓ |
| Parabacteroides_goldsteinii | 0.00111748 | 7.44E-05 | 0.004072 | ↓ |
| Parasutterella_excrementihominis | 0.00630663 | 0.001218 | 0.004072 | ↓ |
| unclassified_Anaeroplasma | 0.00082384 | 3.57E-05 | 0.004072 | ↓ |
| Turicibacter_sanguinis | 0.00196439 | 0.000147 | 0.006502 | ↓ |
| Parabacteroides_distasonis | 0.0066601 | 0.000708 | 0.010165 | ↓ |
| Clostridium_sp._ASF356 | 0.00085207 | 2.35E-05 | 0.01133 | ↓ |
| unclassified_Marvinbryantia | 0.00140961 | 3.65E-05 | 0.01133 | ↓ |
|  |  |  |  |  |

**Supplementary Table 6**

| Differential flora | PBS(Mean) | rEg.P29_B_- (Mean) | *p* value | variation trend |
| --- | --- | --- | --- | --- |
| Bacteroides_caecimuris | 0.00419788 | 0.023957 | 0.000157 | ↑ |
| Turicimonas_muris | 0.00036245 | 0.005127 | 0.000157 | ↑ |
| Lactobacillus_intestinalis | 0.00072776 | 0.004263 | 0.000507 | ↑ |
| unclassified_Alistipes | 0.00098829 | 0.003137 | 0.00194 | ↑ |
| Alistipes_sp._CHKCI003 | 0.00322376 | 0.005887 | 0.004072 | ↑ |
| Lachnospiraceae_bacterium_10_1 | 0.00018242 | 0.002707 | 0.005159 | ↑ |
| Phocaeicola_vulgatus | 0 | 0.001071 | 0.008151 | ↑ |
| Lactobacillus_reuteri | 0.00160352 | 0.004619 | 0.012611 | ↑ |
| Desulfovibrio_fairfieldensis | 0.00798317 | 0 | 0.000157 | ↓ |
| Muribaculum_intestinale | 0.09567714 | 0.008207 | 0.000157 | ↓ |
| Ileibacterium_valens | 0.00623609 | 0 | 0.00067 | ↓ |
| unclassified_unidentified_rumen_bacterium_JW32 | 0.00271421 | 0 | 0.00067 | ↓ |
| Clostridium_sp._ASF356 | 0.00085207 | 0 | 0.008151 | ↓ |
| unclassified_[Eubacterium]_xylanophilum_group | 0.00732485 | 0.000433 | 0.008151 | ↓ |
| unclassified_Enterorhabdus | 0.00050944 | 8.42E-05 | 0.021134 | ↓ |
| Christensenella_minuta | 0.00023475 | 0 | 0.023342 | ↓ |
| Tidjanibacter_massiliensis | 0.00043795 | 0 | 0.023342 | ↓ |
| Turicibacter_sanguinis | 0.00196439 | 0.000363 | 0.023342 | ↓ |
|  |  |  |  |  |

**Supplementary Table 7**

| Differential flora | PBS(Mean) | rEg.P29_T+B_- (Mean) | *P* value | variation trend |
| --- | --- | --- | --- | --- |
| unclassified_[Eubacterium]_ruminantium_group | 8.14E-05 | 0.003655 | 0.003197 | ↑ |
| Firmicutes_bacterium_M10_2 | 0.00178561 | 0.010914 | 0.005159 | ↑ |
| uncultured_Clostridiales_bacterium | 0.00345598 | 0.009537 | 0.006502 | ↑ |
| Clostridium_disporicum | 0 | 0.001149 | 0.008151 | ↑ |
| Clostridium_sp._Clone_27 | 0 | 0.001223 | 0.008151 | ↑ |
| unclassified_Dubosiella | 0.00365552 | 0.025514 | 0.012611 | ↑ |
| uncultured_Bacteroidales_bacterium | 0.04367465 | 0.072892 | 0.01911 | ↑ |
| uncultured_rumen_bacterium | 0.01622958 | 0.049023 | 0.01911 | ↑ |
| Clostridium_sp._Clone_33 | 0 | 0.000288 | 0.023342 | ↑ |
| unclassified_Lachnoclostridium | 9.12E-05 | 0.001677 | 0.023342 | ↑ |
| Desulfovibrio_fairfieldensis | 0.00798317 | 0 | 0.000157 | ↓ |
| Muribaculum_intestinale | 0.09567714 | 0.016058 | 0.000157 | ↓ |
| Ileibacterium_valens | 0.00623609 | 0.000807 | 0.008151 | ↓ |
| Alistipes_sp._CHKCI003 | 0.00322376 | 0.001152 | 0.010165 | ↓ |
| Lachnospiraceae_bacterium_DW22 | 0.00219924 | 0.000571 | 0.023342 | ↓ |
| Parasutterella_excrementihominis | 0.00630663 | 0.002207 | 0.023342 | ↓ |
| Tidjanibacter_massiliensis | 0.00043795 | 0 | 0.023342 | ↓ |
| Bacteroides_thetaiotaomicron | 0.00059265 | 7.29E-05 | 0.025748 | ↓ |
| Clostridium_sp._ASF356 | 0.00085207 | 0.00026 | 0.04125 | ↓ |
| Clostridiales_bacterium_CIEAF_013 | 0.00219738 | 0.000124 | 0.049366 | ↓ |

**Supplementary Table 8**

| Differential flora | PBS(Mean) | rEg.P29 - (Mean) | *P* value | variation trend |
| --- | --- | --- | --- | --- |
| unclassified_Erysipelotrichaceae | 0.00170656 | 0.015444 | 0.000381 | ↑ |
| unclassified_Allobaculum | 0 | 0.002614 | 0.00067 | ↑ |
| unclassified_Colidextribacter | 0.00214138 | 0.008032 | 0.00067 | ↑ |
| Phocaeicola_vulgatus | 0 | 0.00267 | 0.002497 | ↑ |
| Bacteroides_caecimuris | 0.00419788 | 0.01063 | 0.003197 | ↑ |
| Lachnospiraceae_bacterium_DW52 | 3.05E-05 | 0.002051 | 0.003197 | ↑ |
| unclassified_Erysipelatoclostridium | 0.00108855 | 0.008318 | 0.004072 | ↑ |
| unclassified_Muribaculaceae | 0.20300537 | 0.334443 | 0.004072 | ↑ |
| unclassified_Alistipes | 0.00098829 | 0.002823 | 0.005159 | ↑ |
| uncultured_Bacteroidales_bacterium | 0.04367465 | 0.079393 | 0.006502 | ↑ |
| Desulfovibrio_fairfieldensis | 0.00798317 | 0 | 0.000157 | ↓ |
| Muribaculum_intestinale | 0.09567714 | 0.020283 | 0.000157 | ↓ |
| Lachnospiraceae | 0.01582422 | 0.001627 | 0.002497 | ↓ |
| unclassified_Dubosiella | 0.00365552 | 0 | 0.002497 | ↓ |
| Trichinella_pseudospiralis | 0.04411483 | 0.006751 | 0.004072 | ↓ |
| Turicibacter_sanguinis | 0.00196439 | 0.000156 | 0.006502 | ↓ |
| unclassified_GCA_900066575 | 0.00292871 | 0.000596 | 0.014019 | ↓ |
| Tidjanibacter_massiliensis | 0.00043795 | 0 | 0.023342 | ↓ |
| Lachnospiraceae_bacterium_DW22 | 0.00219924 | 0.000585 | 0.028366 | ↓ |
| Colidextribacter_bacterium_ASF500 | 0.0016069 | 0.00023 | 0.04125 | ↓ |

"↑" and "↓" indicate that the intestinal flora is up-regulated and down-regulated
